# Neighborhood Disadvantage, Race and Ethnicity, and Postpartum Depression

**DOI:** 10.1001/jamanetworkopen.2023.42398

**Published:** 2023-11-13

**Authors:** Ticara L. Onyewuenyi, Kelli Peterman, Eve Zaritsky, Miranda L. Ritterman Weintraub, Bria L. Pettway, Charles P. Quesenberry, Nerissa Nance, Ann-Marie Surmava, Lyndsay A. Avalos

**Affiliations:** 1 Obstetrics and Gynecology, Kaiser Permanente Northern California, Oakland; 2 Division of Research, Kaiser Permanente Northern California, Oakland; 3 Graduate Medical Education, Kaiser Permanente Northern California, Oakland; 4 Department of Health Systems Science, Kaiser Permanente Bernard J. Tyson School of Medicine, Pasadena, California

## Abstract

**Importance:**

Postpartum depression (PPD) is a debilitating condition with higher rates among Black individuals. Increasingly, neighborhood disadvantage is being recognized as a contributor to poor health and may be associated with adverse postpartum mental health; however, associations between neighborhood disadvantage, race and ethnicity, and PPD have not been examined.

**Objective:**

To investigate the association between neighborhood disadvantage and PPD and evaluate the extent to which these associations may differ by race and ethnicity.

**Design, Setting, and Participants:**

This population-based cross-sectional study included 122 995 postpartum Kaiser Permanente Northern California members 15 years or older with a live birth between October 7, 2012, and May 31, 2017, and an address in the electronic health record. Analyses were conducted from June 1, 2022, through June 30, 2023.

**Exposures:**

Neighborhood disadvantage defined using quartiles of the Neighborhood Deprivation Index (NDI), a validated census-based socioeconomic status measure; self-reported race and ethnicity ascertained from Kaiser Permanente Northern California electronic health records.

**Main Outcomes and Measures:**

Multivariable Poisson regression was conducted to assess associations between neighborhood disadvantage, race and ethnicity, and a diagnosis of PPD.

**Results:**

Of 122 995 included postpartum individuals, 17 554 (14.3%) were younger than 25 years, 29 933 (24.3%) were Asian, 8125 (6.6%) were Black, 31 968 (26.0%) were Hispanic, 47 527 (38.6%) were White, 5442 (4.4%) were of other race and ethnicity, and 15 436 (12.6%) had PPD. Higher neighborhood disadvantage and race and ethnicity were associated with PPD after covariate adjustment. Compared with White individuals, Black individuals were more likely to have PPD (adjusted relative risk [ARR], 1.30; 95% CI, 1.24-1.37), whereas Asian (ARR, 0.48; 95% CI, 0.46-0.50), and Hispanic (ARR, 0.92; 95% CI, 0.89-0.96) individuals and those identified as having other race and ethnicity (ARR, 95% CI, 0.90; 0.85-0.98) were less likely to have PPD. Associations between NDI and PPD differed by race and ethnicity (likelihood ratio test for interaction, χ^2^_12_ = 41.36; *P *< .001). Among Black individuals, the risk of PPD was the greatest overall and increased with neighborhood disadvantage in a dose-response manner (quartile [Q] 2 ARR, 1.39 [95% CI, 1.13-1.71]; Q3 ARR, 1.50 [95% CI, 1.23-1.83]; Q4 ARR, 1.60 [95% CI, 1.32-1.93]; Cochrane-Armitage test for trend, *P* < .001). Neighborhood disadvantage was associated with PPD among Asian (Q2 ARR, 1.17 [95% CI, 1.04-1.31]; Q3 ARR, 1.20 [95% CI, 1.06-1.35]) and White (Q3 ARR, 1.14 [95% CI, 1.07-1.21]; Q4 ARR, 1.17 [95% CI, 1.09-1.26]) individuals and those of other race and ethnicity (Q3 ARR, 1.34 [95% CI, 1.09-1.63]; Q4 ARR, 1.28 [95% CI, 1.03-1.58]), but the magnitude of risk was lower. Neighborhood disadvantage was not associated with PPD among Hispanic individuals (eg, Q2 ARR, 1.04 [95% CI, 0.94-1.14]; Q3 ARR, 1.00 [95% CI, 0.91-1.10]; Q4 ARR, 0.98 [95% CI, 0.90-1.08]).

**Conclusions and Relevance:**

In this cross-sectional study of postpartum individuals, residing in more disadvantaged neighborhoods was associated with PPD, except among Hispanic individuals. Neighborhood disadvantage may be associated with racial and ethnic differences in postpartum mental health. Geographic targeting of mental health interventions may decrease postpartum mental health inequities.

## Key Points

**Question:**

Is neighborhood disadvantage associated with postpartum depression (PPD), and does the association differ by race and ethnicity?

**Findings:**

In this cross-sectional study of 122 995 postpartum individuals in California, greater neighborhood disadvantage was associated with a higher risk of PPD; the risk was highest among Black individuals. Neighborhood disadvantage was not associated with PPD among Hispanic individuals.

**Meaning:**

Residing in more disadvantaged neighborhoods was associated with poorer postpartum mental health, except among Hispanic individuals; geographic targeting of mental health interventions may decrease postpartum mental health inequities.

## Teaser

This large cross-sectional study of individuals in California evaluates the association between neighborhood disadvantage and postpartum depression and assesses whether any association differs by race and ethnicity among postpartum individuals 15 years or older.

## Introduction

Postpartum depression (PPD) is a serious medical condition impacting between 15% and 20% of postpartum individuals in the US,^1^ with a higher prevalence among Black individuals. Especially when untreated, PPD has lasting impacts on both maternal and child health, such as maternal mortality and morbidity, increased risk for infanticide, poorer maternal-infant attachment, and impaired parenting behaviors.^2,3^ More severe cases of PPD have been associated with an elevated risk of suicide.^2^

An increasing body of evidence suggests neighborhood-level disadvantage, which accounts for area-level educational and employment status, poverty, and housing quality, may be associated with adverse effects on health.^4,5,6,7^ Neighborhoods with high disadvantage are characterized by poor access to good-quality social^8^ and built^9^ environments, creating food deserts and limited walkability, exposure to toxins and pollutants,^9,10^ limited high-quality education and employment opportunities,^11^ crime, and restricted access to health care.^9^ Due to a lack of resources, the deleterious consequences of neighborhood disadvantage may place postpartum individuals at increased risk for depression. An association between higher neighborhood disadvantage and depressive symptomology in nonpregnant populations has been documented^12^; however, research on the association with PPD is lacking.

The compounded stress from limited resources in areas of high disadvantage may be associated with the heightened rates of PPD observed among patients in racial minority groups.^13,14^ It has been posited that the stressors associated with living in a disadvantaged neighborhood are compounded for members of stigmatized minority groups.^15,16^ Black and Hispanic populations are disproportionately affected, as those individuals who develop negative health behaviors in disadvantaged areas experience worse long-term outcomes compared with their White counterparts exhibiting the same behaviors.^17^ However, the association between neighborhood disadvantage, race and ethnicity, and depression is quite limited, particularly in the postpartum period.

The objective of this study was to investigate the association between neighborhood disadvantage and PPD and to evaluate whether the associations differ by race and ethnicity in a large, diverse population-based sample using a validated composite measure for neighborhood disadvantage, the Neighborhood Deprivation Index (NDI). We hypothesized that the risk of PPD would be higher with higher neighborhood disadvantage and differ by race and ethnicity.

## Methods

### Setting

This cross-sectional study was conducted within Kaiser Permanente Northern California (KPNC), a large integrated health care delivery system providing medical care to a large socioeconomically and racially and ethnically diverse population of more than 4.6 million members. All 15 regional service centers (with 44 associated office facilities) have Obstetrics and Gynecology and Behavioral Medicine/Psychiatry Departments. The KPNC members are covered by employee-sponsored insurance plans, the insurance exchange, Medicare, and Medicaid. Coverage is provided for approximately 32.5% of the Northern California population, and characteristics of members are similar to the population living in the Northern California geographic area.^18^ Information on diagnoses, hospitalizations, outpatient visits, and census-level data are maintained within administrative and electronic health records (EHR) databases.^19^ The KPNC institutional review board approved this study and waived the requirement for obtaining informed consent because the study used EHR data only with no participant contact. Study procedures met Health Insurance Portability and Accountability Act requirements and 42 CFR Part 2 regarding medical records. The study followed the Strengthening the Reporting of Observational Studies in Epidemiology (STROBE) guideline for cross-sectional studies.

### Study Design, Population, and Measures

This population-based cross-sectional study included live births between October 7, 2012, and May 31, 2017, to individuals aged 15 years or older with a recorded address in the EHR during either the postpartum period or pregnancy. All data were ascertained from EHR and administrative records of KPNC. We defined PPD by identification of at least 1 depression diagnosis from the *International Classification of Diseases, Ninth Revision *and* Tenth Revision* codes documented in the EHR from the day after delivery and up to 365 days post partum (eAppendix in Supplement 1). As part of standard prenatal care, KPNC members are screened for perinatal depression using the Patient Health Questionnaire-9 (PHQ-9),^20,21,22^ twice during pregnancy and 3 to 10 weeks post partum,^23,24^ and more than 97% of perinatal KPNC members seeking care are screened.^24^ The PHQ-9 is a validated instrument for screening for depression across racial and ethnic groups.^20^ A positive PHQ-9 score (≥10 of 0 to 27) has high sensitivity (>88%) and specificity (>88%) in obstetric patients.^21,22^ The KPNC guidelines include symptom assessment and review of related medical history, and a depression diagnosis is documented in the EHR based on a positive screening score and clinical judgment.^23^

*Neighborhood disadvantage* was defined using the NDI, a census-based socioeconomic index^25^ and standardized to US states in which Kaiser Permanente offers commercial health insurance. The NDI includes socioeconomic status indicators of wealth and income, educational level, occupation, and housing conditions obtained from the American Community Survey. The NDI scores range from −3.6 to 2.8, with higher values indicating greater neighborhood deprivation. The US Census tracts are small and relatively permanent statistical subdivisions of counties and, according to the US Census Bureau, are designed to be homogeneous units with respect to living conditions and sociodemographic characteristics. The current study used the address the individual resided at the most days during the postpartum period, defined from day of delivery to 365 days after delivery. If no address was recorded in the EHR during the postpartum period, the most recent address during pregnancy was used. Each address was matched to the aggregate NDI score for that location, taken from the 2015 American Community Survey. The NDI scores were categorized by quartiles, with quartile cutoffs calculated from NDI scores within the cohort. Each patient was assigned an NDI quartile, with the lowest quartile (Q1) representing the least disadvantaged neighborhood and the highest quartile (Q4) representing the most disadvantaged neighborhood. For comparison purposes, the group with the least neighborhood disadvantage (ie, most advantage; Q1) was selected as the reference category.

Race and ethnicity were ascertained from self-reported EHR data and considered a single construct, defined as Asian or Pacific Islander, Black, Hispanic, White, and other (American Indian or Alaskan Native, multiracial, and unknown). Patients were classified as being Hispanic if they reported Hispanic ethnicity, regardless of race. White race was chosen as the reference category to allow for the evaluation of racial and ethnic disparities and health inequities.

Covariates included maternal age at delivery, prepregnancy body mass index (calculated as weight in kilograms divided by height in meters squared) categorized by obesity status (underweight or normal weight, <25; overweight, 25 to <30; and obese, ≥30), parity (0, 1, 2 or more), and Charlson Comorbidity Index (CCI) score.^26^ The CCI is a tool used to assess the severity of a patient’s comorbid medical conditions. The CCI assigns a numerical score to each comorbidity, and the sum of these scores provide an overall measure of the patient’s comorbidity burden. The higher the score is, the higher the predicted mortality rate.^26^ Calculation of the CCI included diagnosis codes identified in the EHR for the year prior to pregnancy and was categorized as 0 or 1 or higher, or higher. In this sample of postpartum individuals, only 1.4% of women had a CCI score of 2 or higher.

### Statistical Analysis

Categorical variables were reported as percentages, and continuous variables were reported as means and SDs. Bivariate analyses assessed associations between NDI, race and ethnicity, other patient characteristics, and PPD. We used χ^2^ tests to assess associations between categorical variables and PPD, and we used analysis of variance to assess the association between NDI as a continuous variable and race and ethnicity. To assess the associations between NDI, race and ethnicity, and PPD, we used modified Poisson regression analysis.^27^ Multivariable models were adjusted by age at delivery, parity, prepregnancy obesity status, and CCI. Covariates included in the adjusted models were based on statistical significance in bivariate analyses or clinical relevance. To evaluate the presence of effect modification, an interaction term was included in the model between NDI and race and ethnicity, adjusting for age, parity, prepregnancy obesity status, and CCI. Adjusted relative risks (ARRs) were reported with 95% CIs. A 2-sided *P* < .05 was considered statistically significant. Analysis of the data was conducted June 1, 2022, through June 30, 2023, using SAS, version 9.4 (SAS Institute Inc).

## Results

Of 177 003 live births, the first birth per individual that occurred in the study period was included in the present study; thus, 22 345 subsequent births were excluded. A total of 31 663 individuals without complete data (181 missing age; 11 322 missing parity; 16 349 missing body mass index, and 11 missing CCI) were also excluded. Of 122 995 postpartum individuals included, 17 554 (14.3%) were younger than 25 years (mean [SD] age, 30.4 [5.3] years), 24 612 (20.0%) had a minimum of 2 children, 29 933 (24.3%) were Asian, 8125 (6.6%) were Black, 31 968 (26.0%) were Hispanic, 47 527 (38.6%) were White, 5442 (4.4%) were other race and ethnicity, and 15 436 (12.6%) had PPD (Table 1). The percentage of individuals with PPD ranged from 20.8% in the least socioeconomically disadvantaged quartile (NDI Q1) to 27.8% in the highest disadvantaged quartile (NDI Q4). Black (mean [SD] NDI, 0.45 [0.98]) and Hispanic (mean [SD] NDI, 0.29 [0.91]) individuals were more likely to live in areas of higher neighborhood disadvantage compared with Asian (mean [SD] NDI, −0.34 [0.79]), White (mean [SD] NDI, −0.31 [0.71]), and other (mean [SD] NDI, −0.11 [0.85]) individuals (Table 2).

**Table 1.** Characteristics of 122 995 Postpartum Kaiser Permanente Northern California Members Between 2012 and 2017, Overall and by PPD Status

Characteristic	Members, No. (%)			*P* value^a^
	Total (n = 122 995)	PPD (n = 15 436)	No PPD (n = 107 559)	
Race and ethnicity				
Asian	29 933 (24.3)	1893 (12.3)	28 040 (26.1)	<.001
Black	8125 (6.6)	1688 (10.9)	6437 (6.0)	
Hispanic	31 968 (26.0)	4517 (29.3)	27 451 (25.5)	
White	47 527 (38.6)	6624 (42.9)	40 903 (38.0)	
Other^b^	5442 (4.4)	714 (4.6)	4728 (4.4)	
Neighborhood disadvantage				
Q1 (least disadvantaged)	31 583 (25.7)	3213 (20.8)	28 370 (26.4)	<.001
Q2	31 093 (25.3)	3780 (24.5)	27 313 (25.4)	
Q3	30 755 (25.0)	4153 (26.9)	26 602 (24.7)	
Q4 (most disadvantaged)	29 564 (24.0)	4290 (27.8)	25 274 (23.5)	
Age at delivery, y				
<25	17 554 (14.3)	2595 (16.8)	14 959 (13.9)	<.001
25-29	33 776 (27.5)	3980 (25.8)	29 796 (27.7)	
30-34	44 937 (36.5)	5422 (35.1)	39 515 (36.7)	
≥35	26 728 (21.7)	3439 (22.3)	23 289 (21.7)	
Parity				
0	56 194 (45.7)	6383 (41.4)	49 811 (46.3)	<.001
1	42 189 (34.3)	5344 (34.6)	36 845 (34.3)	
≥2	24 612 (20.0)	3709 (24.0)	20 903 (19.4)	
Prepregnancy obesity status				
Underweight or normal	60 599 (49.3)	6300 (40.8)	54 299 (50.5)	<.001
Overweight	33 807 (27.5)	4351 (28.2)	29 456 (27.4)	
Obese	28 589 (23.2)	4785 (31.0)	23 804 (22.1)	
Charlson Comorbidity Index score^c^				
0	103 540 (84.2)	11 979 (77.6)	91 561 (85.1)	<.001
≥1	19 455 (15.8)	3457 (22.4)	15 998 (14.9)	

Abbreviations: PPD, postpartum depression; Q, quartile.

^a^ Values from χ^2^ test of the association.

^b^ Other included American Indian, Alaska Native, multiracial, and unknown.

^c^ The index provides an overall measure of the patient’s comorbidity burden; the higher the score is, the higher the predicted mortality rate.

**Table 2.** Bivariate Associations Between Neighborhood Disadvantage and Race and Ethnicity

Race and ethnicity	NDI, mean (SD)^a^	Members, No. (%)^b^			
		NDI Q1	NDI Q2	NDI Q3	NDI Q4
Asian	−0.34 (0.79)	10 778 (36.0)	8073 (27.0)	6444 (21.5)	4638 (15.5)
Black	0.45 (0.98)	772 (9.5)	1444 (17.8)	2090 (25.7)	3819 (47.0)
Hispanic	0.29 (0.91)	3979 (12.5)	6153 (19.2)	8913 (27.9)	12 923 (40.4)
White	−0.31 (0.71)	14 641 (30.8)	14 077 (29.6)	11 902 (25.1)	6907 (14.5)
Other^c^	−0.11 (0.85)	1413 (26.0)	1346 (24.7)	1406 (25.8)	1277 (23.5)

Abbreviation: NDI, Neighborhood Deprivation Index; Q, quartile.

^a^ NDI scores range from −3.6 to 2.8, with higher values indicating greater neighborhood deprivation. Analysis of variance compared mean NDI scores across different racial and ethnic groups; results indicate *P* < .001.

^b^ The χ^2^ tests compared categorized NDI quartiles (Q1 least disadvantaged, Q4 most disadvantaged) by race and ethnicity; results indicated *P* < .001.

^c^ Other includes American Indian, Alaska Native, multiracial, and unknown.

After adjusting for age at delivery, parity, prepregnancy obesity status, and CCI covariates, compared with White individuals, Black individuals had a 30.0% increased risk of PPD (ARR, 1.30; 95% CI, 1.24-1.37), whereas Asian individuals had a 52.0% decreased risk (ARR, 0.48; 95% CI, 0.46-0.50) and Hispanic individuals had an 8.1% decreased risk (ARR, 0.92; 95% CI, 0.89-0.96) (Table 3). Additionally, compared with individuals in neighborhoods with the lowest disadvantage (NDI Q1), individuals living in areas of higher disadvantage had an increased risk of PPD (Q2 ARR, 1.09 [95% CI, 1.04-1.14]; Q3 ARR, 1.15 [95% CI, 1.10-1.20]; Q4 ARR, 1.14 [95% CI, 1.09-1.20]). The interaction term for race and ethnicity and NDI was statistically significant (likelihood ratio test for interaction, χ^2^_12_ = 41.36; *P *< .001).

**Table 3.** Relative Risk for Associations between Neighborhood Disadvantage, Race and Ethnicity, and PPD

Variable	PPD, RR (95% CI)	
	Crude (N = 122 995)	Adjusted (N = 122 995)^a^
Neighborhood disadvantage (reference is Q1)		
Q2	1.12 (1.08-1.18)	1.09 (1.04-1.14)
Q3	1.20 (1.15-1.26)	1.15 (1.10-1.20)
Q4	1.23 (1.17-1.28)	1.14 (1.09-1.20)
Race and ethnicity (reference is White)		
Asian	0.45 (0.43-0.48)	0.48 (0.46-0.50)
Black	1.41 (1.34-1.48)	1.30 (1.24-1.37)
Hispanic	0.97 (0.93-1.00)	0.92 (0.89-0.96)
Other	0.93 (0.86-0.99)	0.90 (0.85-0.98)

Abbreviations: PPD, postpartum depression; Q, quartile; RR, relative risk.

^a^ Adjusted for age at delivery, parity, prepregnancy obesity status, and Charlson Comorbidity Index score.

The associations between NDI and PPD differed when stratified by race and ethnicity (Figure). Among Black individuals, the risk of PPD increased in a dose-response manner from 39.0% to 60.0% as NDI increased (NDI Q2 ARR, 1.39 [95% CI, 1.13-1.71]; NDI Q3 ARR, 1.50 [95% CI, 1.23-1.83]; NDI Q4 ARR, 1.60 [95% CI, 1.32-1.93]; Cochrane-Armitage test for trend, *P* < .001). Among Asian individuals, when compared with NDI Q1, a similar increased risk of PPD was found across the higher quartiles of NDI (Q2 ARR, 1.17 [95% CI, 1.04-1.31]; Q3 ARR, 1.20 [95% CI, 1.06-1.35]), although it did not reach statistical significance for neighborhoods in the highest NDI quartile (Q4 ARR, 1.10 [95% CI, 0.96-1.27]). Among White individuals, higher NDI was associated with increased risk of PPD for the 2 most disadvantaged quartiles (Q3 ARR, 1.14 [95% CI, 1.07-1.21]; Q4 ARR, 1.17 [95% CI, 1.09-1.26]). A similar pattern was found for individuals of other race and ethnicity (Q3 ARR, 1.34 [95% CI, 1.09-1.63]; Q4 ARR, 1.28 [95% CI, 1.03-1.58]). Neighborhood disadvantage was not associated with PPD among Hispanic individuals (eg, Q2 ARR, 1.04 [95% CI, 0.94-1.14]; Q3 ARR, 1.00 [95% CI, 0.91-1.10]; Q4 ARR, 0.98 [95% CI, 0.90-1.08]).

**Figure.**
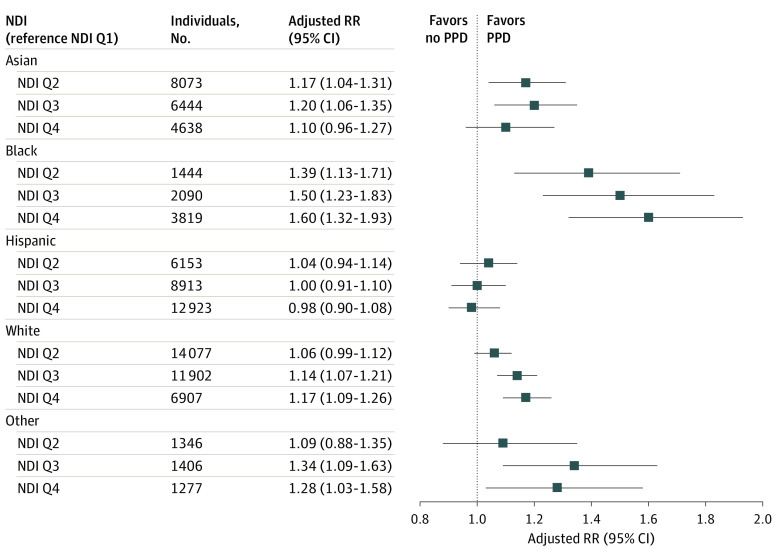
Association Between Neighborhood Disadvantage and Postpartum Depression (PPD), Stratified by Race and Ethnicity NDI indicates Neighborhood Deprivation Index; Q, quartile; RR, relative risk.

## Discussion

In this large, diverse cross-sectional study of 122 995 postpartum individuals covering urban and rural areas in Northern California, our findings indicated neighborhood disadvantage as an independent factor associated with PPD. Furthermore, these disparities varied by racial and ethnic group. Among Hispanic individuals there was no association between neighborhood disadvantage and PPD. However, higher neighborhood disadvantage was associated with PPD for Asian, Black, and White individuals and those of other races and ethnicities. Of significance, not only was the risk of PPD associated with neighborhood disadvantage strongest among Black individuals, but we documented a dose-response association. Additionally, Black individuals overall had the highest risk of PPD, whereas Asian and Hispanic individuals had a reduced risk of PPD compared with White individuals. Black individuals were also more likely to live in neighborhoods of higher disadvantage. Collectively, the study results suggest neighborhood disadvantage is associated with Black-White inequities in postpartum mental health outcomes. This study is among the first, to our knowledge, to assess the complex interplay between the mental health status of individuals during the postpartum period, race and ethnicity, and socioeconomic disadvantage using neighborhood-level characteristics, as opposed to individual-level measures. Findings from this study contribute to a better understanding of perinatal mental health inequities.

These study findings provide new information relating to Black individuals and specifically to neighborhood disadvantage; Black individuals from neighborhoods with greater deprivation have poorer health outcomes, including mental health.^13,14^ The association between higher neighborhood disadvantage with higher rates of PPD among Black individuals is likely multifactorial and complex. Systemic factors and discriminatory practices, such as redlining and housing discrimination, inequitable access to education, employment discrimination, disproportionate rates of incarceration, and health disparities, add a tremendous burden to the normal stressors of life.^28,29^ Lifestyle factors such as food-intake patterns, sleep status, exercise, and physical activity can significantly affect PPD.^30^ In general, individuals living in neighborhoods without access to healthy food options, safe outdoor activities, or community support are deprived of opportunities for self-care and psychological well-being.

By contrast, neighborhood disadvantage was not associated with PPD among postpartum Hispanic individuals. A study in a more general population reported similar results.^31^ Additionally, Hispanic individuals had a decreased risk of PPD compared with White individuals. Further research needs to be conducted to understand these findings, but these results lend support to the social cohesion hypothesis, which refers to the capacity of a society to ensure the welfare of all of its members and minimize disparities.^32^

Among Asian individuals in the present study, neighborhood disadvantage was associated with PPD as disadvantage increased, although not at the highest level of disadvantage. Previous research^33^ among older Chinese adults noted that neighborhood social cohesion was positively associated with lower levels of psychological distress and higher levels of life satisfaction. Our study highlights the need for exploration of these associations by Asian racial and ethnic subgroups, and future studies are needed to investigate whether higher social cohesion is limited to the most disadvantaged neighborhoods among Asian individuals.

This study contributes to the growing body of evidence showing associations between neighborhood disadvantage and adverse health outcomes and behaviors.^4,5,6,7^ Our findings highlight the need for partnerships with community organizations to implement both community- and individual-level interventions aimed at improving postpartum mental health, with special attention paid to the Black community. These interventions may help address the inequities observed in postpartum mental health. Collaboration with community organizations to provide psychosocial interventions delivered by trained community peers could help to overcome the myriad of barriers that prevent access to mental health care in disadvantaged neighborhoods. Clinical-level interventions also have a role to play in improving these disparities. Nationwide implementation of universal depression screening during postpartum visits, culturally appropriate screening tools, and health insurance coverage that offers access to mental health services may all be effective policy initiatives to reduce the financial burden of seeking care and to decrease the stigma associated with PPD. Policies to improve housing and neighborhoods to help reverse the damage done from prior red-lining policies may help to decrease PPD disparities by NDI. The multigenerational consequences of PPD, including adverse developmental, cognitive, and mental health outcomes in children, underscore the importance of multimodal interventions that address the broader social determinants of health to enhance the overall well-being of communities.^3,34,35^

### Strengths and Limitations

This study has several strengths. This analysis is among the first and largest population-based studies to use clinical data to examine the complex interplay between neighborhood disadvantage, race and ethnicity, and postpartum mental health. We used a comprehensive composite measure to assess the complexity of neighborhood disadvantage as opposed to using a single-metric neighborhood socioeconomic status characteristic or individual-level socioeconomic indicator (eg, income or educational attainment). Race and ethnicity were based on self-report. The use of data from KPNC’s large universal perinatal depression screening program, in which more than 97% of perinatal patients are screened,^23,24^ and the study’s use of postpartum diagnoses data ascertained from the EHR reduce the chance of recall bias and outcome misclassification. Furthermore, this study included a large and heterogenous sample, which encompassed both rural and urban regions, and a 5-year study period.

This study has limitations. First, given the cross-sectional study design, we cannot infer causal associations. Second, data on individual-level socioeconomic characteristics were not included in the EHR. Given that individual-level socioeconomic factors have been associated with PPD, future research should determine whether individual-level or neighborhood-level factors are more salient contributors to PPD. Third, this study was limited to insured members of a large, integrated health care delivery system and may not be generalizable to individuals without insurance. Additionally, although this study included a large sample size, the population was limited to Northern California and therefore may not be generalizable to other areas in the United States.

## Conclusions

This cross-sectional study found that inferior postpartum mental health outcomes were associated with residence in neighborhoods with greater disadvantage. Furthermore, the findings suggest that neighborhood disadvantage may be associated with the observed Black-White inequities in postpartum mental health. Additionally, neighborhood disadvantage was not associated with PPD among Hispanic individuals. The prevalence of PPD among Hispanic individuals did not differ by neighborhood disadvantage status and highlights the importance of future investigations to understand factors that could be contributing to this adverse health condition. Study findings suggest that the implementation of social and public health policies that prioritize investment in disadvantaged neighborhoods may improve postpartum mental health outcomes and reduce health disparities in the US, particularly among Black individuals.

## Notes

Supplement 1. **eAppendix.** List of International Statistical Classification of Diseases and Related Health Problems (ICD) Depression Diagnosis Codes Included in the Analysis

Supplement 2.
Data Sharing Statement
