# Supplementary material for: Neighborhood Disadvantage, Race and Ethnicity, and Postpartum Depression
Source: JAMA Netw Open. 2023 Nov 13;6(11):e2342398. doi: 10.1001/jamanetworkopen.2023.42398 (PMC10644210; doi:10.1001/jamanetworkopen.2023.42398)
Supplement: Supplement 1. — eAppendix. List of International Statistical Classification of Diseases and Related Health Problems (ICD) Depression Diagnosis Codes Included in the Analysis [file jamanetwopen-e2342398-s001.pdf]

Supplementary Online Content

Onyewuenyi TL, Peterman K, Zaritsky E, et al. Neighborhood disadvantage, race and ethnicity, and postpartum depression. *JAMA Netw Open*. 2023;6(11):e2342398. doi:10.1001/jamanetworkopen.2023.42398

eAppendix. List of International Statistical Classification of Diseases and Related Health Problems (ICD) depression diagnosis codes included in the analysis.

This supplementary material has been provided by the authors to give readers additional information about their work.

© 2023 Onyewuenyi TL et al. *JAMA Network Open*.

eAppendix. List of International Statistical Classification of Diseases and Related Health Problems (ICD) Depression Diagnosis Codes Included in the Analysis.

**ICD-9 Depression diagnosis codes**	296.20 - 296.25<br>296.30 - 296.35<br>298.0<br>300.4<br>309.0<br>309.1<br>311<br>648.4
**ICD-10 Depression diagnosis codes**	F33.9<br>F34.1<br>F43.21<br>F43.23<br>F53.0<br>O90.6<br>O99.34

© 2023 Onyewuenyi TL et al. *JAMA Network Open*.
