# Supplementary material for: Neighborhood Disadvantage, Race and Ethnicity, and Postpartum Depression
Source: JAMA Netw Open. 2023 Nov 13;6(11):e2342398. doi: 10.1001/jamanetworkopen.2023.42398 (PMC10644210; doi:10.1001/jamanetworkopen.2023.42398)
Supplement: Supplement 2. — Data Sharing Statement [file jamanetwopen-e2342398-s002.pdf]

Data Sharing Statement

Onyewuenyi. Neighborhood Disadvantage, Race and Ethnicity, and Postpartum Depression. *JAMA Netw Open*. Published November 13, 2023. doi:10.1001/jamanetworkopen.2023.42398

Data
Data available: No
